# Peripheral nerve stimulation targeting the trochanteric branch of the femoral nerve for refractory greater trochanteric pain syndrome followed by dorsal root ganglion stimulation: A case report and mechanistic perspective

**DOI:** 10.1016/j.inpm.2026.100770

**Published:** 2026-05-07

**Authors:** Charles A. Odonkor, David A. Karpe

**Affiliations:** a Department of Orthopedics and Rehabilitation, Yale University School of Medicine, Yale New Haven Hospital, 47 College Street, New Haven, CT, 06520, United States; b University of New England College of Osteopathic Medicine, 716 Stevens Avenue, Portland, ME, 04103, United States

**Keywords:** Greater trochanteric pain syndrome, Peripheral nerve stimulation, Dorsal root ganglion stimulation, Neuromodulation, Refractory hip pain, Case report

## Abstract

**Introduction:**

Greater trochanteric pain syndrome (GTPS) is a common cause of chronic lateral hip pain and may remain refractory despite pharmacologic therapy, physical therapy, and injection-based interventions. While radiofrequency ablation targeting the trochanteric branch of the femoral nerve has been described as a potential treatment, the use of peripheral nerve stimulation targeting this sensory branch remains limited.

**Case report:**

We report a case of refractory GTPS in a 40-year-old woman treated with peripheral nerve stimulation (PNS) followed by dorsal root ganglion stimulation (DRG).

**Intervention and outcome:**

A temporary 60-day PNS system targeting the trochanteric branch of the femoral nerve resulted in complete resolution of hip pain during the treatment period. Following recurrence of lateral hip pain after lead removal, persistent longstanding low back and buttock pain prompted DRG stimulation to address the patient's broader preexisting pain distribution. Permanent implantation of right L1–L2 and bilateral S1 DRG leads resulted in approximately 75% reduction in both residual lumbosacral pain and recurrent lateral hip pain, with durable functional improvement.

**Conclusion:**

This case highlights the trochanteric branch of the femoral nerve as a potential neuromodulation target for refractory GTPS. Peripheral nerve stimulation may provide a minimally invasive, targeted approach for lateral hip pain, while DRG stimulation may offer broader dermatomal coverage in patients with overlapping pain distributions. Further study is warranted.

## 1 Introduction

Greater trochanteric pain syndrome (GTPS) is a common cause of chronic lateral hip pain, affecting approximately 15 percent of women and 6 percent of men in the general population [1]. Imaging and sonographic studies have demonstrated that most symptomatic cases are related to gluteal tendinopathy and other peri-trochanteric soft-tissue pathology as opposed to isolated inflammation of the trochanteric bursa [2,3]. Patients typically present with localized lateral hip pain that worsens with ambulation, prolonged sitting, or lying on the affected side. In individuals with persistent symptoms, structural abnormalities such as gluteal tendon tears, iliotibial band pathology, and peri-trochanteric calcifications have been reported [4,5].

Initial management focuses on conservative strategies, including activity modification, structured physical therapy, nonsteroidal anti-inflammatory medications and corticosteroid injection of the trochanteric bursa [6]. When symptoms persist, several minimally invasive interventions may be considered. Extracorporeal shockwave therapy has demonstrated improvement in pain and functional outcomes in randomized trials [7], and systematic reviews suggest it may provide benefit compared with conservative therapy alone [8]. Platelet-rich plasma injections have also been explored as a treatment option, with randomized trials demonstrating improvements in pain and functional outcomes in some patient populations [9]. However, other analyses report variable effectiveness among conservative management strategies, indicating the heterogeneity of outcomes in this condition [10]. Similarly, systematic reviews comparing biologic and surgical therapies have reported mixed results depending on patient selection and disease severity [11].

Percutaneous ultrasound-guided tenotomy has also been recognized as a minimally invasive option, particularly in patients with degenerative gluteal tendon or iliotibial band pathology, and observational studies have reported desirable outcomes following this approach [12,13]. For patients who fail conservative and minimally invasive therapies, surgical treatment may be considered. Arthroscopic and endoscopic procedures such as bursectomy, iliotibial band release, and abductor tendon repair have demonstrated improvements in pain and functional outcomes in appropriately selected patients [[14], [15], [16]]. In some cases, concomitant intra-articular pathology, including labral tears and chondral lesions, may play a role in persistent symptoms and alter treatment decisions [17].

In parallel with these treatment strategies, neuromodulation has increasingly been explored as an approach for the management of chronic pain syndromes. By targeting peripheral or spinal neural structures, neuromodulation aims to modulate nociceptive signaling pathways and improve functional outcomes. Peripheral nerve stimulation (PNS) has demonstrated benefit across a range of conditions, including chronic pain involving the lower extremities [[18], [19], [20]]. While prior interventional studies have explored radiofrequency ablation of the trochanteric branch of the femoral nerve, the use of PNS targeting this branch has not been well described. To our knowledge, sequential use of PNS followed by dorsal root ganglion (DRG) stimulation for persistent GTPS has not previously been reported.

## 2 Case report

A 40-year-old woman with a history of obesity status post gastric sleeve surgery presented with longstanding low back pain and severe right-sided lateral hip pain consistent with GTPS. Her hip pain had progressively worsened over several years and had begun to significantly limit ambulation, sleep, and daily activities. Prior MRI and ultrasound of the hip demonstrated gluteus medius and minimus tendinopathy at their greater trochanteric insertions, thickening of the tensor fascia lata and iliotibial band along with an anterior labral tear and peri-trochanteric edema. Plain radiographs demonstrated mild degenerative changes consistent with hip osteoarthritis. Prior treatment included structured physical therapy, corticosteroid injections, regenerative treatments with tendonotomy, shockwave therapy and platelet-rich plasma injections targeting the gluteal tendon attachments and fascia. She also tried multiple oral analgesic medications including nonsteroidal anti-inflammatory drugs, anticonvulsants, antidepressants, and opioid medications, without adequate or durable relief. Despite these interventions, she continued to report persistent pain and functional impairment with a baseline average daily numeric rating score of 8/10.

**Fig. 1:**
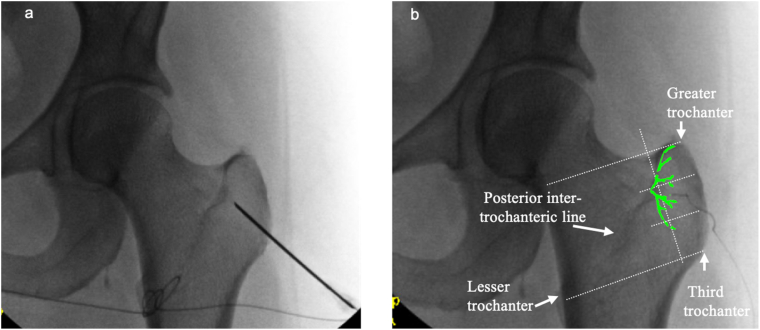
Fluoroscopic guided peripheral nerve stimulation targeting the trochanteric branch of the femoral nerve. Fluoroscopic image demonstrating peripheral nerve stimulation lead placement targeting the trochanteric branch of the femoral nerve adjacent to the greater trochanteric region. a. Stimulating needle electrode at nerve target. b. SPRINT PNS microlead. Green outline highlights the trajectory of the trochanteric branch of the femoral nerve along the posterior greater trochanter. (For interpretation of the references to colour in this figure legend, the reader is referred to the Web version of this article.)

The patient had longstanding low back, buttock, and posterior thigh pain in addition to severe focal lateral hip pain. Because the lateral hip pain was the dominant and most functionally limiting symptom, she first underwent a 60-day PNS treatment targeting the greater trochanteric branch of the right femoral nerve in February 2024 (Fig. 1). Although this resulted in complete but temporary relief of the lateral hip pain, she continued to experience persistent low back and buttock symptoms. Given this overlapping pain distribution and corresponding imaging findings, including L3–L4 disc protrusion and facet arthropathy, she subsequently underwent lumbar epidural steroid injection, medial branch blocks, and sacroiliac joint injection later in 2024 to evaluate and treat potential coexisting pain generators. Despite these interventions, persistent lateral hip pain remained, supporting a refractory peri-trochanteric pain source. The sequence of diagnostic evaluation, interventional procedures, and neuromodulation therapies is summarized in Table 1.

**Table 1:** Clinical timeline of evaluation, interventions, and outcomes.

Time Period	Evaluation/Event	Intervention	Outcome
January 2024	Initial consultation after orthopedic referral for refractory GTPS. Examination demonstrated greater trochanteric tenderness, hip abductor weakness, positive FABER and single-leg stance tests, and mild Trendelenburg gait. Prior PRP, percutaneous tendonotomy, and shockwave therapy had been ineffective.	Treatment options discussed, including peripheral nerve stimulation (PNS) and radiofrequency ablation (RFA).	Patient elected to proceed with temporary PNS.
February 2024	Procedural visit and early follow-up	Temporary 60-day PNS implanted targeting the trochanteric branch of the femoral nerve.	Procedure completed without complications. At 2-week follow-up, the patient reported approximately 80% reduction in lateral hip pain without device-related adverse events.
April 2024	Completion of 60-day treatment period	End of temporary PNS therapy	Complete (100%) relief of lateral hip pain with improved ambulation and sleep.
July 2024	Recurrence of symptoms with persistent low back and buttock pain	Diagnostic sacroiliac joint injection	No meaningful pain relief.
August–December 2024	Persistent overlapping pain despite prior interventions; lumbar MRI demonstrated facet arthropathy and L3–L4 central disc protrusion.	Lumbar epidural steroid injections, medial branch blocks/facet interventions, and piriformis injections.	No sustained improvement in lateral hip, buttock, or low back pain.
Late 2024	Reassessment of treatment strategy	Options discussed included repeat PNS, RFA, and DRG stimulation to provide broader coverage of the patient's overlapping pain distribution.	Patient elected evaluation for DRG stimulation. Permanent PNS was considered but not available within the health system.
January 2025	Pre-implant evaluation	Psychological screening for neuromodulation candidacy	Cleared for DRG therapy.
March 2025	DRG trial	Trial stimulation with right L2 and right S1 DRG leads	Approximately 80% improvement in lateral hip pain and substantial improvement in buttock and low back pain, although anterior hip/groin pain remained incompletely covered.
March 2025	Permanent implantation procedure	Permanent DRG system implanted with right L1–L2 and bilateral S1 leads to expand coverage of both hip and lumbosacral pain.	Procedure completed without complications.
April 2025	Early postoperative follow-up	Device programming and monitoring	100% relief of lateral hip pain, approximately 60% improvement in low back pain, and approximately 80% improvement in buttock and posterior thigh pain.
September 2025	Six-month follow-up	Continued DRG therapy	Approximately 75% overall pain reduction with improved sleep and functional capacity.

### 2.1 Procedural targeting technique

**Fig. 2:**
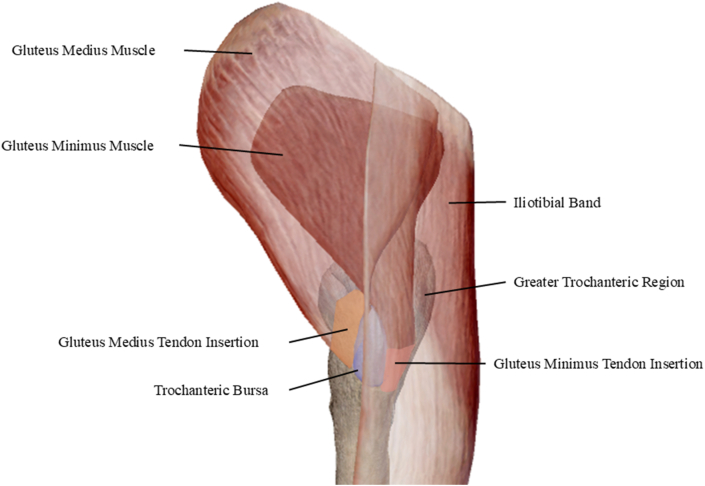
Anatomy of the greater trochanteric region and peri trochanteric structures associated with GTPS. Anatomical illustration of the greater trochanteric region demonstrating the gluteus medius and gluteus minimus tendon insertions, the greater trochanter, and surrounding peri-trochanteric structures commonly implicated in GTPS. Base anatomy generated using Visible Body Human Anatomy Atlas (Visible Body, Boston, MA), with additional labeling by the authors.

The anatomic structures commonly involved in GTPS include the gluteus medius and gluteus minimus tendons, the greater trochanteric bursa, and the surrounding peri-trochanteric soft tissues (Fig. 2). Fluoroscopic targeting was guided by previously described anatomical landmarks along the posterior aspect of the greater trochanter, where the trochanteric branch of the femoral nerve courses along the periosteum [21]. Cadaveric anatomical studies show that this sensory branch travels posteriorly toward the greater trochanter after passing between the quadratus femoris and inferior gemellus tendons before arborizing over the periosteal surface of the trochanter and surrounding bursae [22]. Targeting this region allows modulation of sensory input supplying the peri-trochanteric structures implicated in GTPS.

With the patient positioned prone and under anterior–posterior fluoroscopic guidance, the posterior aspect of the greater trochanter was identified. The optimal stimulation zone corresponds to the proximal two-thirds of the posterior greater trochanter, defined via the intersection of two fluoroscopic reference lines extending from the superior trochanter–femoral neck junction and from the femoral shaft (third trochanter) toward the lesser trochanter, as previously described [22]. This region was selected because the trochanteric branch of the femoral nerve lies directly along the periosteum in this location, allowing reliable targeting with fluoroscopic guidance.

After infiltration of the skin entry site with 1% lidocaine, a 20-gauge blunt introducer electrode was advanced toward the posterior–lateral aspect of the greater trochanter until periosteal contact was achieved. The periosteal surface acts as an osseous backstop, allowing accurate positioning while avoiding the major gluteal tendon insertions and adjacent musculotendinous structures. The selected zone lies inferior to the primary insertion of the gluteus medius and lateral to the quadratus femoris tendon, allowing neuromodulation of sensory fibers in a relatively safe and reproducible location.

Anatomical studies suggest variability in the terminal arborization of the trochanteric branch as it spreads across the periosteal surface of the greater trochanter [21]. Targeting the proximal posterior trochanteric region, therefore, allows capture of multiple terminal sensory branches supplying the peri-trochanteric tissues. Histologic analyses additionally support the suitability of this target, demonstrating that the trochanteric branch of the femoral nerve is predominantly sensory, consisting largely of unmyelinated fibers innervating the periosteum and surrounding bursae [21].

Final lead positioning was confirmed using intraoperative sensory stimulation mapping (Fig. 1a and b). The external pulse generator was secured according to manufacturer guidelines. Postoperatively, the patient used a handheld wireless controller to titrate stimulation to a comfortable sensory level within the device's programmed parameter range (intensity scale 0–100, corresponding to approximately 0.2–30 mA with pulse widths between 10 and 200 μs). Stimulation was permitted on an as-needed basis for up to 24 hours per day.

During and at the end of the 60-day treatment period, the patient reported complete resolution of lateral hip pain with marked improvement in mobility. However, three months following lead removal, she experienced recurrence of lateral hip pain while continuing to have longstanding low back, buttock, and posterior thigh pain. Recrudescence of her index pain and overlapping lumbosacral pain suggested involvement of both peripheral and segmental pain pathways beyond the original focal generator. Given the persistence of both recurrent lateral hip pain and the patient's longstanding lumbosacral and buttock pain, DRG stimulation was pursued to provide broader dermatomal coverage. A DRG trial was performed with placement of two leads: one at the right L2 DRG to target the dominant lateral hip pain, and one at the right S1 DRG to address concurrent lumbosacral and buttock symptoms. During the trial, the patient reported substantial improvement in posterior buttock, low back, and lateral hip pain; however, anterior hip and groin pain remained inadequately covered. Based on this incomplete anterior coverage, the decision was made to expand targeting to include the right L1 DRG for implant.

**Fig. 3:**
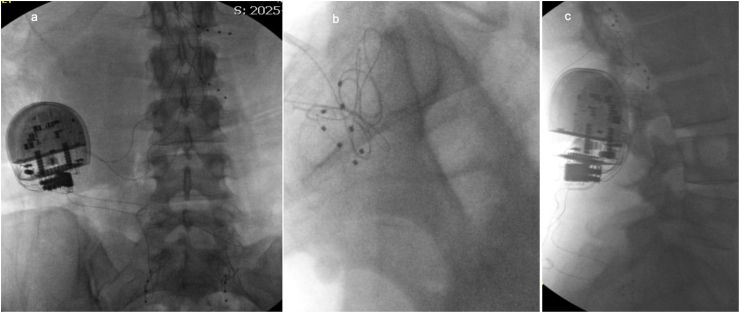
Fluoroscopic guided DRG stimulation lead placement at right L1–L2 and bilateral S1 levels. Fluoroscopic image demonstrating DRG stimulation lead placement at the right lumbar L1 and L2 DRG and bilateral sacral S1 DRG leads, respectively. a. Anterior-Posterior view of S1 and right L1,L2 DRG leads. b. Lateral view of S1 DRG leads. c. Lateral view of L1 and L2 DRG leads.

Following a successful trial, permanent implantation of right L1–L2 and bilateral S1 DRG leads was performed (Fig. 3a–c) to optimize coverage of both anterior/lateral hip and lumbosacral pain distributions. Notably, the patient reported that inclusion of S1 stimulation contributed to a broader and more global sense of pain relief, particularly for posterior distribution symptoms. With respect to programming, two stimulation programs were utilized, with the patient predominantly using a single primary program approximately 90% of the time based on symptom relief and comfort. As an example, her preferred program used 90% of the time included right L1 and L2 stimulation at 20 Hz, 200 μs pulse width, and 0.1 mA amplitude, with right S1 and left S1 at 20 Hz, 200 μs pulse width, and 0.1 mA, whereas the less preferred program parameters were right L1 and L2 (10 Hz, 170 μs pulse width and 0.1mA) and left S1 and right S1 (14 Hz, 130 μs pulse width and 0.1mA). At follow-up, the patient reported approximately 75% reduction in overall pain, improved restorative sleep, and meaningful gains in functional capacity, with a patient global impression of change score greater than 6 (“very much improved”). No procedural complications or device-related adverse events were observed.

## 3 Discussion

Management of refractory GTPS remains challenging, particularly in patients who continue to experience substantial pain and functional limitation despite conservative therapy. Several minimally invasive interventions have been described for refractory disease, including extracorporeal shockwave therapy, platelet-rich plasma injections, percutaneous tenotomy, and radiofrequency ablation targeting the trochanteric branch of the femoral nerve [[7], [8], [9], [10], [11], [12], [13],^22^,^23^]. Surgical treatment may also be considered as a last resort in carefully selected patients with structural pathology [[14], [15], [16], [17]]. A summary of commonly described treatment approaches is provided in Table 2.

**Table 2:** Summary of treatment strategies for refractory GTPS.

Treatment Category	Intervention	Evidence Summary	Typical Clinical Role
Conservative therapy	Activity modification, physical therapy, nonsteroidal anti-inflammatory medications, and corticosteroid injection of the trochanteric bursa	Conservative measures and corticosteroid injection are considered first-line treatment for GTPS and may provide short-term improvement in pain and function [6]	Initial management for most patients with GTPS before consideration of more advanced interventions
Conservative therapy	Extracorporeal shockwave therapy	Randomized controlled trials and meta-analyses demonstrate improvement in pain and functional outcomes in patients with GTPS [7,8]	Considered after failure of physical therapy and corticosteroid injections but before invasive procedures
Biologic therapy	Platelet-rich plasma injections	Randomized trials and systematic reviews demonstrate improvement in pain scores in some patients with refractory symptoms, although results across studies are mixed [[9], [10], [11]]	Option for patients with persistent symptoms who have not responded to corticosteroid injections
Minimally invasive procedures	Percutaneous ultrasound-guided tenotomy	Observational studies demonstrate improvement in pain and functional outcomes with continued benefit at one year follow up [12,13]	Used for patients with suspected gluteal tendinopathy who fail conservative and injection-based therapies
Interventional pain procedures	Radiofrequency ablation targeting the trochanteric branch of the femoral nerve	Early interventional studies exhibit pain reduction in patients with refractory GTPS [22,23]	Considered for patients who fail injections and prefer to avoid surgical intervention
Neuromodulation	PNS	Systematic reviews and clinical guidelines support the use of peripheral nerve stimulation for focal neuropathic and chronic pain syndromes [[18], [19], [20]]	May serve as a minimally invasive neuromodulation option for focal pain distributions and can function as a diagnostic and therapeutic step
Neuromodulation	DRG stimulation	Randomized clinical trials validate improved treatment success compared with traditional spinal cord stimulation for certain neuropathic pain syndromes [24]	Considered for patients with overlapping or broader chronic pain distributions involving the hip, buttock, or lower extremity after failure of less invasive therapies
Surgical management	Arthroscopic or endoscopic treatment including bursectomy, iliotibial band release, or abductor tendon repair	Surgical case series and scoping reviews showed improvement in pain and functional outcomes in patients with structural pathology or persistent symptoms [[14], [15], [16], [17]]	Reserved for patients with structural abnormalities or persistent symptoms after failure of conservative and minimally invasive therapies

In the present case, the patient had persistent lateral hip pain despite physical therapy, medications, corticosteroid injection, regenerative interventions, and percutaneous tendonotomy performed elsewhere. Temporary PNS was selected as a minimally invasive neuromodulation strategy targeting the trochanteric branch of the femoral nerve after shared decision-making regarding procedural alternatives. Although radiofrequency ablation was considered, the patient's marked weight loss and relatively thin, soft-tissue envelope over the greater trochanter raised concern for cutaneous dysesthesia or thermal injury with lesioning near the periosteal surface. In this context, temporary PNS offered a reversible, non-destructive approach.

Accurate targeting of the trochanteric branch remains an important procedural consideration. Because this branch is small and not reliably visualized with ultrasound, fluoroscopic targeting of the posterior greater trochanter using established osseous landmarks may provide a practical and reproducible strategy [[21], [22], [23]]. In this patient, complete resolution of lateral hip pain during the 60-day treatment period is consistent with the clinical relevance of this target and suggests that the trochanteric sensory branch was a meaningful contributor to the index pain generator.

Following lead removal, the patient experienced recurrence of lateral hip pain while continuing to have longstanding low back, buttock, posterior thigh, and lumbosacral symptoms. Because the pain phenotype extended beyond the focal lateral hip region, DRG stimulation was pursued to provide broader dermatomally targeted coverage. Trial stimulation at right L2 and right S1 provided good coverage of posterior buttock, low back, and lateral hip pain but did not adequately address the anterior hip and groin. This prompted addition of the right L1 at permanent implantation. The final configuration of right L1-L2 and bilateral S1 leads was therefore selected to address the patient's combined right anterior/lateral hip and bilateral lumbosacral pain distributions.

The selected DRG targets were based on pain distribution rather than strict peripheral nerve root origin alone. In this case, L1-L2 targeting was used to address groin, lateral hip, and lateral thigh pain consistent with upper lumbar dermatomal coverage, whereas S1 stimulation was added to address concomitant lumbosacral, buttock, and posterior thigh pain. Although some dermatomal overlap may exist, S1 was not the primary target for GTPS-related pain. The patient's long-term use of both upper lumbar and sacral leads, along with her report that S1 stimulation enhanced global relief for posterior distribution symptoms, supports the functional relevance of this broader targeting strategy in her specific presentation.

From a mechanistic standpoint, both PNS and DRG stimulation may reduce pain by modulating abnormal sensory signaling, although the exact mechanisms cannot be determined from a single case [[25], [26], [27], [28], [29], [30], [31]]. In this patient, temporary PNS appeared to effectively modulate focal peri-trochanteric pain, whereas later DRG stimulation allowed broader coverage of an overlapping dermatomal pain distribution. These interventions should therefore be understood as distinct treatments applied at different stages of the patient's clinical course rather than as a cumulative or synergistic neuromodulation sequence.

This case highlights several clinically relevant points. First, the trochanteric branch of the femoral nerve may represent a useful neuromodulation target in selected patients with refractory GTPS. Second, a strong response to temporary PNS may help support the contribution of focal peri-trochanteric sensory pathways to the patient's pain [[23], [25]]. Third, when patients have overlapping pain distributions extending beyond the original focal lateral hip pain, DRG stimulation may offer a means of addressing overlapping dermatomal pain distributions involving the lateral hip and lumbosacral region. As a single-case report, these observations are descriptive and should not be interpreted as establishing efficacy or a standard treatment pathway. Further study is needed to better define patient selection, optimal targeting, and the role of neuromodulation in refractory GTPS.

## 4 Conclusion

GTPS can remain highly disabling in patients who fail conservative and injection-based therapies. This case highlights the potential role of anatomically targeted neuromodulation in managing refractory lateral hip pain. PNS directed at the trochanteric branch of the femoral nerve may offer a minimally invasive approach to modulate peri-trochanteric nociceptive input and may also function as a practical diagnostic adjunct in select patients. In contrast, DRG stimulation provides more proximal, dermatomally targeted modulation, allowing coverage of both lateral hip and concomitant lumbosacral pain distributions [[32], [33]].

While limited by its single-case design, this report suggests that neuromodulation strategies tailored to pain distribution may warrant further investigation in patients with refractory GTPS.

## Ethics statement

This case report was conducted in accordance with the principles of the Declaration of Helsinki. Written informed consent was obtained from the patient for participation and publication of this case report and accompanying images. Institutional review board approval was not required for a single patient case report in accordance with institutional policy.

## Outside disclosures

Unrelated to this submission, Dr. Charles A. Odonkor is a consultant for Boston Scientific and SPR Pain Relief.

## Author contributions

Charles A. Odonkor conceptualized the case report, supervised the clinical aspects of the study, performed the literature review and edited the manuscript. David A. Karpe finalized the literature review and drafted the complete manuscript. All authors reviewed and approved the final manuscript.

## Funding

This research did not receive any specific grant from funding agencies in the public, commercial, or not-for-profit sectors.

## Competing interests

The authors declare that they have no relevant competing interests.
